# Supplementary material for: The impact of farmers’ digital literacy on farmers’ enthusiasm for grain cultivation: Findings from the “Double Hundred and Thousand” farmers in Jiangxi province
Source: PLoS One. 2025 Dec 26;20(12):e0334437. doi: 10.1371/journal.pone.0334437 (PMC12742759; doi:10.1371/journal.pone.0334437)
Supplement: S1 Data — (XLSX) [file pone.0334437.s001.pdf]

Data Sharing Declaration

Statement issued by: Jiangxi Agricultural University "Three Rural Issues Research Center"

Related paper: 'The Impact of Farmers' Digital Literacy on Farmers' Enthusiasm for Grain Cultivation: Findings from the' Double Hundred and Thousand 'Farmers in Jiangxi Province' (Manuscript Number: ONE-D-25-33960R1; DOI: 10.1371/journal. pone. 0334437)

1.Data ownership and compliance statement

All data used in this research paper were independently collected, organized, and analyzed by the "Three Rural Issues Research Center" of Jiangxi Agricultural University, and the ownership of the data belongs exclusively to the center. The entire process of data collection, processing, and storage strictly follows the data sharing policy of PLOS ONE journal, national scientific research data management standards, and academic ethics requirements. There are no legal restrictions, ethical disputes, or intellectual property disputes that hinder data disclosure and sharing.

2.Specific commitments for data sharing

To ensure the reproducibility of research and the dissemination of academic achievements, our center agrees to publicly share the "minimum data-set" that supports the core conclusions of the paper, including raw research data, metadata, and key data required for analysis. The relevant data has been placed in Excel and can be attached to the paper. The shared data format conforms to academic standards and fully corresponds to the research conclusions of the paper, making it easy for peer verification and secondary research use.

3.Data Usage Standards and Responsibility Statement

When researchers use this data for academic research purposes, they need to cite this paper and the data source in a standardized manner, and clearly indicate that the data belongs to the 'Three Rural Issues Research Center' of Jiangxi Agricultural University. Our center is responsible for the authenticity and accuracy of the data, and is not liable for any derivative results arising from third-party use of the data. The data

扫描全能王 创建

sharing period is long-term public. If you have any questions about data access or use, please contact: Wang Zhipeng; Contact email: jxnddyxf@jxau.edu.cn and wzhipeng1991@163.com ; Contact phone number: 0791-3828107/0791-3828014.

Jiangxi Agricultural University "Three Rural Issues Research Center

November 14, 2025

江西农业大学 三农问题研究中心

扫描全能王 创建

数据共享声明

声明出具单位：江西农业大学 “三农” 问题研究中心

关联论文：《The Impact of Farmers' Digital Literacy on Farmers' Enthusiasm for Grain Cultivation: Findings from the "Double Hundred and Thousand" Farmers in Jiangxi Province》（Manuscript Number：PONE-D-25-33960R1；DOI：10.1371/journal.pone.0334437）

一、数据归属与合规性说明

本论文研究所用全部数据，均由江西农业大学 “三农” 问题研究中心独立采集、整理及分析生成，数据所有权归该中心独家所有。数据收集、处理及存储全过程严格遵循《PLOS ONE》期刊数据共享政策、国家科研数据管理规范及学术伦理要求，无法律限制、伦理争议或知识产权纠纷阻碍数据公开共享。

二、数据共享具体承诺

为保障研究的可重复性与学术成果传播，本中心同意公开共享支撑论文核心结论的“最小数据集”，包括原始调研数据、元数据及分析所需关键数据。相关数据已放在 Excel 中，可作为附件放在论文中。共享数据格式符合学术通用标准，与论文研究结论完全对应，便于同行验证及二次研究使用。

三、数据使用规范与责任声明

科研人员基于学术研究目的使用该数据时，需规范引用本论文及数据来源，明确标注“数据归属江西农业大学‘三农’问题研究中心”。本中心对数据的真实性、准确性负责，不对第三方使用数据产生的衍生结果承担责任。 数据共享期限为长期公开，若有数据访问、使用相关疑问，可联系：王智鹏；联系邮箱：jxnddyxf@jxau.edu.cn 和 wzhipeng1991@163.com；联系电话：0791-3828107/0791-3828014。

江西农业大学 “三农” 问题研究中心

2025 年 11 月 14 日

江西农业大学 三农问题研究中心

扫描全能王 创建
