# Supplementary material for: The impact of farmers’ digital literacy on farmers’ enthusiasm for grain cultivation: Findings from the “Double Hundred and Thousand” farmers in Jiangxi province
Source: PLoS One. 2025 Dec 26;20(12):e0334437. doi: 10.1371/journal.pone.0334437 (PMC12742759; doi:10.1371/journal.pone.0334437)
Supplement: Attachment — Submitted filename: Response to Reviewers.docx [file pone.0334437.s003.docx]

**The Impact of Farmers’ Digital Literacy on Farmers’ Enthusiasm for Grain Cultivation: Findings from the “Double Hundred and Thousand” Farmers in Jiangxi Province**

**Response to Reviewers**

We sincerely thank the editor and the reviewers for their precious time and invaluable comments, which were of great help in revising the manuscript. We have modified the manuscript accordingly and the detailed corrections are listed below point by point. We have used the same comment numbering that the reviewers used. The revised parts are shown in red in the resubmitted manuscript.

**Response to Reviewer # 1**

1.Title Optimization

The original title is overly lengthy. “Double Hundred and Thousand” refers to the survey project name rather than core concepts, which could be relocated to the data description section. Suggested revision: “Research on the Impact of Digital Literacy on Farmers’ Enthusiasm for Grain Cultivation: An Empirical Analysis Based on Double-Cropping Rice Cultivation in Jiangxi Province”.

Thank you for pointing out this! After reviewing the entire text, reconsider and revise the title of this article to “The Impact of Farmers’ Digital Literacy on Farmers’ Enthusiasm for Grain Cultivation: Findings from the ‘Double Hundred and Thousand’ Farmers in Jiangxi Province”.

2.Introduction Transition

The section from “the importance of food security” to “the role of digital literacy” lacks a logical bridge. Add transitional sentences to create a smoother connection between ideas.

Thank you for your valuable suggestion! We have added transitional sentences to the original text to ensure smooth transitions; the specific additions can be found in the red-marked sections of the manuscript.

3.Literature Gap Justification

The statement that “academic research on the impact of digital literacy on farmers’ enthusiasm for grain cultivation remains limited” needs to be substantiated by citing emerging studies (even those with conflicting conclusions) to precisely situate this paper’s contributions.

Thank you for pointing out this! The statement that “academic research on the impact of digital literacy on farmers’ enthusiasm for grain cultivation remains limited” lacks sufficient precision and rigor in its description; therefore, we propose revising that section to read: Moreover, the existing research has achieved certain results in the field of farmers’ grain production behavior and digital literacy effect, and it is of certain value to further explore the influence mechanism of farmers’ digital literacy on farmers’ enthusiasm for grain cultivation.

4.Introduction Streamlining

The introduction section could be appropriately condensed while maintaining clear logic and smooth transitions between ideas.

Thank you for your valuable suggestion! The introduction section has been appropriately condensed to ensure clear logic and natural transitions; the specific revisions can be found in the red-marked sections of the manuscript.

5.Mechanism Analysis Depth

The mechanism analysis could be more nuanced. For example, beyond information access, does digital literacy enhance farmers’ market forecasting ability, risk tolerance, or operational management capacity when promoting farmland transfer? How does this differ from mere land transfer platform usage?

Thank you for pointing out this! The text has explicitly stated that: “Farmers with high digital literacy typically leverage digital platforms to proactively access emerging trends in agriculture, rural areas, and farmer-related domains, breaking information barriers and translating external informational advantages into agricultural management capabilities and improving production efficiency.” The specific responses are presented as follows:

**I. Core Role of Farmland Transfer-in and Empowerment Mechanism of Digital Literacy**

Farmers’ digital literacy enhances enthusiasm for grain cultivation by accelerating farmland transfer-in, with the core logic being: digitally skilled farmers can more efficiently utilize digital tools to break information barriers, convert external information into agricultural management capabilities, thereby expanding operational scale and improving production efficiency. This mechanism can be analyzed through three dimensions: market prospect judgment capacity, risk resilience, and operational management capacity.

**(I) Market Prospect Judgment Capacity: Transforming Information Advantages into Decision-Making Advantages**

**1.Information Acquisition and Price Forecasting**

Leveraging digital platforms, farmers with high digital literacy can access real-time agricultural product prices, supply-demand data, and policy information, using data analysis to predict market trends. For instance, by integrating agrarian market data with meteorological information, farmers can accurately assess the profit expectations of double-cropping rice cultivation, avoiding “double-cropping to single-cropping” concealed farmland abandonment. A Jiangxi Province study also reveals that digitally skilled farmers excel in using online sales platforms to expand marketing channels, adjusting crop structures through price forecasting and directly curbing concealed farmland abandonment (Chen et al., 2025).

**2.Policy Responsiveness and Opportunity Capture**

Digital literacy enables farmers to swiftly respond to government policies such as “prime farmland for grain use” and agricultural subsidies. For example, accessing land transfer subsidy information via government platforms reduces farmland transfer-in costs and secures policy dividends.

**(II) Risk Resilience: Digital Tools Mitigate Production and Market Risks**

**1.Production Risk Management**

**Disease Monitoring and Early Warning**

Utilizing remote sensing data, meteorological information, and AI models, digitally skilled farmers can predict Fusarium head blight and rice blast risks 7 days in advance, reducing pesticide use and crop losses.

**Digital Financial Support**

Through privacy-computing platforms integrating land contract data with farmer credit information, digitally literate farmers gain easier access to low-interest loans, alleviating financial constraints during land transfer and enhancing risk resilience.

**2.Market Risk Hedging**

**Diversified Sales Channels**

Digital literacy enables farmers to engage in contract farming via e-commerce platforms, reducing reliance on traditional buyers. For example, blockchain technology for product traceability enhances brand premium capacity and stabilizes revenue expectations.

**(III) Operational Management Capacity: Scaling and Efficiency Enhancement**

**1.Optimized Large-Scale Operation**

**Land Transfer Efficiency**

Digitally skilled farmers use digital platforms to rapidly match land transfer-out and transfer-in parties, reducing information asymmetry. And digital finance reduces land transfer costs, facilitating operational scale expansion.

**Resource Integration Capacity**

Leveraging digital twin technology to create virtual farms enables real-time monitoring of equipment, crops, and markets. For instance, 3D modeling optimizes irrigation and fertilization plans, lowering per-unit production costs.

**2.Technology Adoption and Innovation**

**Green Production Technology Application**

Digitally literate farmers are more likely to adopt water-saving irrigation and smart agricultural machinery. Research shows digital literacy enhances double-cropping rice efficiency by promoting agricultural socialization services (Chen et al., 2025).

**II. Conclusion**

Farmers’ digital literacy accelerates farmland transfer-in and boosts grain cultivation enthusiasm through three pathways: accurate market prospect judgment, dynamic production risk control, and sustained operational efficiency optimization. This mechanism not only validates Hypothesis H2 but also reveals the multi-dimensional empowerment effects of digital literacy in agricultural modernization.

[1] Chen J H, Chen J, Qiu H L. The Impact of Digital Literacy on Implicit Farmland Abandonment ——A Study Based on Farmers’ “from Double to Single” Grain Cultivation. Journal of Huazhong Agricultural University (Social Sciences Edition).2025;01:15-30. https://10.13300/j.cnki.hnwkxb.2025.01.003

6.Model Equation Presentation

In the model explanation section, avoid inserting mathematical formulas in the main text to prevent inconsistent paragraph spacing.

Thank you for your valuable suggestion! The model selection section has had its formatting of formulas and symbols standardized; the specific revisions can be found in the red-marked sections of the manuscript.

7.Measurement Validity Concerns

Table 1 shows “digital technology literacy” measured by only one item (digital technology adoption). This may raise validity concerns and requires justification.

Thank you for pointing out this! The entropy method typically involves steps such as data normalization, calculating the proportion of each sample under each indicator, computing information entropy, and determining weights. Each indicator is processed independently, and theoretically, the weight of each indicator is determined based on its own level of variation, unrelated to the number of other indicators. Therefore, even if a certain dimension contains only one item, as long as the data for that item is processed correctly, its weight should be determined by the information capacity of the item itself, rather than the number of items in its group. Consequently, differences in the number of items across dimensions in the entropy method (e.g., “digital technology literacy” having only one item) generally do not directly affect the final calculation results.

8.Instrumental Variable Validity

The “municipal mobile phone penetration rate” correlates strongly with regional economic development, which may directly affect grain cultivation enthusiasm through non-farm opportunities and infrastructure. Although “village economic development level” is controlled, potential omitted variable bias remains. The exogeneity of this instrumental variable and its limitations need more thorough discussion. Consideration of more micro-level instruments (e.g., village/community digital infrastructure) is advised.

Thank you for your valuable suggestion! Next, we will thoroughly discuss the rationale behind the relevance and exogeneity of the instrumental variable “municipal mobile phone penetration rate”.

Theoretical Justification for the Instrumental Variable:

First, the municipal mobile phone penetration rate satisfies the relevance condition. Mobile phones are the primary terminal for accessing digital information in rural China. A higher regional mobile phone penetration rate indicates better development of local mobile network infrastructure, which lowers the cost of access and use for residents. This creates an objective environment conducive to farmers’ exposure to and engagement with digital tools (e.g., mobile apps, online payment, information search). Consequently, farmers in municipalities with higher penetration rates are more likely to develop digital skills, indicating a strong correlation between the IV and the endogenous variable (farmers’ digital literacy).

Second, the municipal mobile phone penetration rate plausibly satisfies the exclusion restriction. The penetration rate, as a macro-level indicator of infrastructure, does not directly determine an individual farmer’s decision to plant double-cropping rice or their planting area. Its influence on cultivation behavior is primarily channeled through its effect on shaping the individual’s digital literacy. We control for other potential confounding factors at the village level (e.g., economic development level, distance to the county seat) that might be correlated with both infrastructure and agricultural decisions. Furthermore, the variation in mobile phone penetration across municipalities is largely determined by historical investments in telecommunications infrastructure and regional topography, factors that can be considered exogenous to individual farmers’ current grain production choices.

The first-stage regression results (Table 4) confirm a statistically significant positive correlation between the IV and farmers’ digital literacy. The F-statistic exceeds the critical value of 10, robustly rejecting the hypothesis of a weak instrument. Therefore, the “municipal mobile phone penetration rate” is a valid and strong instrumental variable.

9.Heterogeneity Analysis Robustness

In the education-level heterogeneity analysis, grouping “junior high school or below” vs “senior high school or above” is standard but requires reporting sample distribution (Table 7 shows N=730 vs N=191). The stability of results for the smaller sample group (senior high school or above) should be discussed. Similar sample size concerns apply to cooperative participation groups (non-members N=711 vs members N=210). Interaction term regression could enhance analysis.

Thank you for pointing out this! The specific revisions are presented as follows:

For the heterogeneity analysis of educational attainment, we divided the sample into three groups: low educational attainment group, medium educational attainment group, and high educational attainment group, and conducted grouped regression. The grouped regression results are presented in the following table, with a relatively balanced distribution of sample sizes across the groups.

**Regression results by educational attainment groups.**

| **Variable** | **Low education group** | | **Medium education group** | | **High education group** | |
| --- | --- | --- | --- | --- | --- | --- |
|  | Double-cropping rice cultivation decision | Double-cropping rice cultivation area | Double-cropping rice cultivation decision | Double-cropping rice cultivation area | Double-cropping rice cultivation decision | Double-cropping rice cultivation area |
| Farmers’ digital literacy | 0.362  (0.481) | 0.631  (0.518) | 0.642^*^  (0.363) | 0.831^**^  (0.414) | 1.451^***^  (0.547) | 1.523^***^  (0.515) |
| Control variables | Controlled | Controlled | Controlled | Controlled | Controlled | Controlled |
| Pseudo R^2^ | 0.0917 | 0.1207 | 0.1071 | 0.1339 | 0.1549 | 0.1651 |
| N | 336 | 336 | 394 | 394 | 191 | 191 |

Notes: *, **, and *** denote significance at the 10%, 5%, and 1% levels, respectively; values in parentheses are standard errors.

For the heterogeneity analysis of cooperative membership, due to the significant disparity in sample sizes between the two groups in the subgroup regression, an interaction term approach was initially adopted but yielded unsatisfactory results. Therefore, we revised the heterogeneity analysis framework to focus on the level of household part-time farming. Specifically, we categorized the sample into four groups: pure agricultural households, Primary part-time farming households, Secondary part-time farming households, and non-agricultural households, and conducted grouped regression. The results are presented in the following table, with a relatively balanced distribution of sample sizes across the groups.

**Regression results by** **level of** **household part-time farming subgroups**

| **Variable** | **Pure** **agricultural households** | | **Primary part-time farming households** | |
| --- | --- | --- | --- | --- |
|  | Double-cropping rice cultivation decision | Double-cropping rice cultivation area | Double-cropping rice cultivation decision | Double-cropping rice cultivation area |
| Farmers’ digital literacy | -0.165  (0.905) | 0.814  (0.799) | 0.737  (0.679) | 0.577  (0.758) |
| Control variables | Controlled | Controlled | Controlled | Controlled |
| Pseudo R^2^ | 0.2143 | 0.1238 | 0.1602 | 0.1350 |
| N | 122 | 122 | 159 | 159 |

| **Variable** | **Secondary part-time farming households** | | **Non-****agricultural households** | |
| --- | --- | --- | --- | --- |
|  | Double-cropping rice cultivation decision | Double-cropping rice cultivation area | Double-cropping rice cultivation decision | Double-cropping rice cultivation area |
| Farmers’ digital literacy | 0.627^*^  (0.372) | 0.961^**^  (0.377) | 1.352^**^  (0.555) | 0.739^*^  (0.404) |
| Control variables | Controlled | Controlled | Controlled | Controlled |
| Pseudo R^2^ | 0.0712 | 0.0871 | 0.0905 | 0.0451 |
| N | 406 | 406 | 234 | 234 |

Notes: *, **, and *** denote significance at the 10%, 5%, and 1% levels, respectively; values in parentheses are standard errors.

10.Heterogeneity Dimension Expansion

The heterogeneity analysis could incorporate additional dimensions for more comprehensive exploration.

Thank you for your valuable suggestion! In the heterogeneity analysis section, we have incorporated an additional analysis of the level of household part-time farming. The specific results are detailed in Amendment 9.

11.Policy Recommendation Precision

The policy suggestions section should better leverage findings from heterogeneity analysis to propose more targeted recommendations.

Thank you for pointing out this! In the policy recommendations section, based on the heterogeneity analysis results, an additional item has been included, with the specific added content outlined as follows.

4. Implement differentiated digital empowerment strategies based on the level of household part-time farming. Accurately identify household types through a dynamic management database to enable targeted interventions. For pure agricultural households: Promote lightweight digital tools, provide “one-on-one” hands-on training via village service stations, and support micro-digital upgrades to enhance basic production efficiency. For primary part-time farming households: Deliver integrated “digital + agri-skills” training by embedding smart agriculture modules into vocational programs, and concurrently develop seasonal smart farming services to mitigate seasonal labor constraints. For secondary part-time farming households and non-agricultural households: Establish regional digital platforms integrating grain market prices and supply chain finance, and promote market-oriented models like cloud-based farm management and contract farming to strengthen data-driven cultivation decisions.

**Response to Reviewer # 2**

1. Clarify the Study’s Original Contributions

The current introduction does not clearly articulate the novelty of the paper. The authors are encouraged to list 2–3 explicit contributions at the end of the Introduction, e.g.: The construction of a five-dimensional digital literacy index tailored to rural China; Identification of farmland transfer, machinery services, and green technology as mediating mechanisms; Heterogeneity analysis by education level and cooperative membership.

Thank you for pointing out this! The current introduction does clearly articulate the novelty of the paper. The specific revisions are presented as follows:

Building on this, in order to improve farmers’ enthusiasm for grain cultivation and promote agricultural production and income increase, this study takes the traditional double-cropping rice production area of Jiangxi Province as the research area, and deeply explores the influence mechanism and action path of farmers’ digital literacy on farmers’ enthusiasm for grain cultivation. The following contributions are made:

First, the evaluation system of farmers’ digital literacy suitable for rural China is constructed from five dimensions: digital media literacy, digital information literacy, digital social literacy, digital financial literacy and digital technology literacy. Second, it identifies farmland transfer-in, agricultural machinery socialization services, and adoption of green grain production technologies as intermediary mechanisms. Third, the heterogeneity analysis is carried out according to educational attainment and the level of household part-time farming. Fourth, grounded in theoretical and empirical findings, targeted recommendations are proposed to elevate farmers’ digital literacy and amplify cultivation motivation, delivering actionable strategies to enhance agricultural income and safeguard national food security.

2. Define “Enthusiasm for Grain Cultivation” More Precisely

The term “enthusiasm” is used repeatedly but is operationalized via behavior (whether or not to grow double-cropping rice). Since “enthusiasm” typically refers to emotional or attitudinal variables, consider rephrasing or providing a clearer construct definition (e.g., “measured enthusiasm through behavioral proxies such as crop choice and cultivation area”).

Thank you for your valuable suggestion! we have adjusted the formulation of the dependent variable “farmers’ enthusiasm for grain cultivation” to enhance its precision; the specific revision is outlined below.

The dependent variable in this study is farmers’ enthusiasm for grain cultivation, measured through behavioral proxies such as double-cropping rice cultivation decisions and cultivation area.

3. Improve Figure 1 with Caption and English Terms

The theoretical framework figure lacks an informative caption and currently mixes Chinese-style conceptualization with English labeling. Revise the figure with: All variable names in English; A caption that explains arrows and variables (e.g., “This diagram illustrates the hypothesized mechanisms linking digital literacy to grain cultivation behavior.”)

Thank you for pointing out this! We have renamed the title of Figure 1 to “The mechanism of the effect of farmers’ digital literacy on farmers’ enthusiasm for grain cultivation” to make the information in Figure 1 clearer.

4. Expand the Explanation of the Empirical Model

While the use of Probit and Tobit models is appropriate, the manuscript lacks clarity about variable definitions and the reasoning for model choice. Include a section that explicitly defines all variables and symbols in the model equations to enhance transparency.

Thank you for your valuable suggestion! In the “3 Data Source, Variable Setting and Model Selection” section of this paper, a detailed explanation of the empirical model has already been provided. Now we focus on elaborating the rationale for variable definition and model selection, with the specific content outlined as follows.

**Variable Definition**

| **Variable type** | **Variable name** | **Definition and coding** |
| --- | --- | --- |
| Dependent variables | Double-cropping rice cultivation decision | 0=No; 1=Yes |
|  | Double-cropping rice cultivation area (log) | Sown area (mu), logarithmic transformation |
| Core explanatory variable | Farmers’ digital literacy | Index calculated via entropy method |
| Control variables | Gender | 0=Female; 1=Male |
|  | Age | Actual age (years) |
|  | Educational attainment | 1=No formal education; 2=Primary school; 3=Junior high; 4=Senior high; 5=College+ |
|  | Health status | 1=Very poor; 2=Poor; 3=Fair; 4=Good; 5=Excellent |
|  | Satisfaction with grain purchase prices | 1=Very dissatisfied; 2=Dissatisfied; 3=Neutral; 4=Satisfied; 5=Very satisfied |
|  | Number of agricultural laborers | Number of family members engaged in farming |
|  | Social capital | Social interaction expenditure (Log) |
|  | Cultivated land area | Total farmland area (mu) |
|  | Paddy field fertility | 1=Very poor; 2=Poor; 3=Fair; 4=Good; 5=Excellent |
|  | Cooperative membership | 0=No; 1=Yes |
|  | Land transfer behavior | 0=No; 1=Yes |
|  | Agricultural income share | Percentage of household income from farming (%) |
|  | Village economic development level | 1=Very low; 2=Low; 3=Medium; 4=High; 5=Very high |
|  | Distance to county seat | Kilometers from village to county center |
| Mechanism Variables | Farmland transfer-in | Transferred-in farmland area (mu)， logarithmic transformation |
|  | Agricultural machinery socialization services | Number of mechanized services adopted (0–6) |
|  | Adoption of green grain production technologies | Number of sustainable practices adopted (0–7) |

**Model Selection**

The dependent variable is farmers’ enthusiasm for grain cultivation, where the double-cropping rice cultivation decision is a binary variable (0/1). As the Probit model is suitable for binary dependent variables and following the approach of Zhu M and Yang R , the Probit model is selected for empirical analysis. Accordingly, the following model is constructed:

$\Pr o(\text{decision}_{i}=1)=\Phi(\alpha_{1}digital_{i}+\beta_{1}X+\mu_{1i})$ (1)

Where *digital_i_* is the core explanatory variable, X represents control variables (including individual characteristics, household characteristics, and village characteristics), *decision_i_* is the dependent variable (1 = adoption of double-cropping rice; 0 = non-adoption), and *µ_1i_* denotes the random error term.

For the double-cropping rice cultivation area, the Probit model is no longer applicable. Since only cultivation area data for adopters are observable (non-adopters’ values are truncated to zero), a Tobit model is constructed for regression:

$Ln\text{（}area_{i}+1\text{）}=\alpha_{2}digital_{i}+\beta_{2}X+\mu_{2i}$ (2)

Where the dependent variable *area_i_* is the logarithm of the double-cropping rice cultivation area, and other variables align with Equation (1).

5. Strengthen Justification of Instrumental Variable

The choice of “municipal mobile phone penetration rate” as an instrument for digital literacy needs stronger theoretical and empirical support. Authors should: Explain why this IV is correlated with digital literacy; Justify why it is not directly related to cultivation behavior; Cite prior literature or data on mobile infrastructure variation.

Thank you for pointing out this! To mitigate endogeneity caused by omitted variables and reverse causality, this study employs the instrumental variable (IV) approach. Drawing on the research of Li et al. [1] and established economic theory, we utilize the “municipal mobile phone penetration rate” as an IV for farmers’ digital literacy.

Theoretical Justification for the Instrumental Variable:

First, the municipal mobile phone penetration rate satisfies the relevance condition. Mobile phones are the primary terminal for accessing digital information in rural China. A higher regional mobile phone penetration rate indicates better development of local mobile network infrastructure, which lowers the cost of access and use for residents. This creates an objective environment conducive to farmers’ exposure to and engagement with digital tools (e.g., mobile apps, online payment, information search). Consequently, farmers in municipalities with higher penetration rates are more likely to develop digital skills, indicating a strong correlation between the IV and the endogenous variable (farmers’ digital literacy).

Second, the municipal mobile phone penetration rate plausibly satisfies the exclusion restriction. The penetration rate, as a macro-level indicator of infrastructure, does not directly determine an individual farmer’s decision to plant double-cropping rice or their planting area. Its influence on cultivation behavior is primarily channeled through its effect on shaping the individual’s digital literacy. We control for other potential confounding factors at the village level (e.g., economic development level, distance to the county seat) that might be correlated with both infrastructure and agricultural decisions. Furthermore, the variation in mobile phone penetration across municipalities is largely determined by historical investments in telecommunications infrastructure and regional topography, factors that can be considered exogenous to individual farmers’ current grain production choices.

The first-stage regression results (Table 4) confirm a statistically significant positive correlation between the IV and farmers’ digital literacy. The F-statistic exceeds the critical value of 10, robustly rejecting the hypothesis of a weak instrument. Therefore, the “municipal mobile phone penetration rate” is a valid and strong instrumental variable.

[1] Li J, Peng Z. Impact of digital villages on agricultural green growth based on empirical analysis of Chinese provincial data. Sustainability.2024;16(21):9590. https://doi.org/10.3390/su16219590

6. Standardize Tables and Improve Formatting

Capitalize variable names and avoid overly technical abbreviations; Report confidence intervals in addition to standard errors; Align table styles with PLOS ONE formatting standards (e.g., using bold for significant variables, consistent decimals).

Thank you for your valuable suggestion! We have standardized the table formatting and optimized the layout, as evidenced by the red-highlighted sections in the manuscript.

7. Eliminate Redundancies and Vague Phrasing

Phrases such as “significantly positive effect on enthusiasm” are repeated frequently. Use more varied and precise language, e.g., “Digital literacy significantly increases the likelihood of adopting double-cropping rice” or “enhances the scale of cultivation.”

Thank you for pointing out this! We have revised the repetitive phrasing “significantly positive effect on enthusiasm” using more nuanced and precise language, as indicated in the red-marked sections of the manuscript.

8. Summarize Mechanism Findings More Clearly in Conclusion

The current conclusion summarizes the baseline results well but underplays the mediation analysis. Add a brief sentence such as: “Among the three mediating pathways, farmland transfer-in had the strongest effect, indicating that access to land remains a key channel for enhancing production behavior.”

Thank you for your valuable suggestion! We have added a brief sentence “Among the three mediating pathways, farmland transfer-in had the strongest effect, indicating that access to land remains a key channel for enhancing production behavior.”

9. Prioritize Policy Recommendations

The policy section is comprehensive but could benefit from prioritization. For example, recommend first improving digital infrastructure, then farmer training, and finally green technology dissemination, according to feasibility and impact.

Thank you for pointing out this! For the policy recommendations section, we have structured them in a logical sequence that corresponds to the theoretical analysis, with detailed and appropriate content. Firstly, comprehensively accelerate the improvement of rural farmers’ digital literacy levels by improving hardware infrastructure and strengthening farmer training programs. Secondly, accelerate farmland transfer-in, expand agricultural operational scale, and promote agricultural machinery socialization services. Thirdly, popularize green grain production technologies. Finally, implement differentiated digital empowerment strategies based on the level of household part-time farming.

10. Deepen Discussion on Digital Divide

The “Further Discussion” section raises valuable points about digital inequality. Consider adding specific mitigation suggestions such as: “To avoid marginalizing elderly or low-education farmers, policies could introduce rural ‘digital envoys’ to provide peer-based guidance in digital farming.”

Thank you for your valuable suggestion! We have supplemented the “Further Discussion” section with mitigation measures addressing perspectives on digital inequality, as detailed below.

Although the study confirms the positive role of digital literacy, potential contradictions in policy implementation require vigilance: Deepening heterogeneity in the digital divide: Elderly farmers with lower education levels may face “technological displacement” due to digital skill deficiencies, necessitating safeguards against exacerbated group disparities during technology promotion. To avoid marginalizing elderly or low-education farmers, policies could introduce rural “digital envoys” to provide peer-based guidance in digital farming Structural imbalances in agricultural machinery service markets: Current machinery cooperatives predominantly focus on plowing/harvesting stages, while high-end services such as drone-based pest control and intelligent monitoring remain insufficient, potentially limiting the realization of digital technology dividends. In response to this problem, high-end agricultural machinery equipment such as intelligent monitoring can be included in the scope of national agricultural machinery purchase subsidies, and the proportion of subsidies can be increased to directly reduce the procurement costs of farmers and cooperatives. Adaptability challenges in green technology promotion: As far as breeding technology is concerned, centralized breeding may not fully meet the needs of small farmers for varieties or variety groups that are more adaptable to the local environment, and participatory and decentralized methods and models need to be explored.

**Response to Reviewer #3**

The title needs revisions. The authors can consider the suggestion below

The Impact of Farmers’ Digital Literacy on Farmers’ Enthusiasm for Grain Cultivation: Findings from the “Double Hundred and Thousand” Farmers in Jiangxi Province

Thank you for pointing out this! we have revised the title to read: The Impact of Farmers’ Digital Literacy on Farmers’ Enthusiasm for Grain Cultivation: Findings from the “Double Hundred and Thousand” Farmers in Jiangxi Province.

3 Data Source, Variable Setting and Model Selection

What was the study design? What impact design was used?

Thank you for your valuable suggestion! The specific responses are as follows:

**1. Study Design**

The research adopts a cross-sectional observational design with structured survey data, characterized by:

**(1) Data Source & Sampling**

**Region:** Jiangxi Province (China’s core double-cropping rice region, representing 25.4% of national early rice area).

**Sampling: Stratified random sampling:**

Stratification: 24 counties → 3 tiers (high/medium/low economic development) → 8 counties/tier → 3 townships/county → 3 villages/township.

**Units:** 10 households/village → Initial 2,160 households → Final 921 valid observations after filtering.

**Time:** 2023 survey (“Double Hundred and Double Thousand” project by Jiangxi Agricultural University).

**(2) Variables Operationalization**

| **Variable type** | **Variable name** | **Definition and coding** |
| --- | --- | --- |
| Dependent variables | Double-cropping rice cultivation decision | 0=No; 1=Yes |
|  | Double-cropping rice cultivation area (log) | Sown area (mu), logarithmic transformation |
| Core explanatory variable | Farmers’ digital literacy | Index calculated via entropy method |
| Control variables | Gender | 0=Female; 1=Male |
|  | Age | Actual age (years) |
|  | Educational attainment | 1=No formal education; 2=Primary school; 3=Junior high; 4=Senior high; 5=College+ |
|  | Health status | 1=Very poor; 2=Poor; 3=Fair; 4=Good; 5=Excellent |
|  | Satisfaction with grain purchase prices | 1=Very dissatisfied; 2=Dissatisfied; 3=Neutral; 4=Satisfied; 5=Very satisfied |
|  | Number of agricultural laborers | Number of family members engaged in farming |
|  | Social capital | Social interaction expenditure (Log) |
|  | Cultivated land area | Total farmland area (mu) |
|  | Paddy field fertility | 1=Very poor; 2=Poor; 3=Fair; 4=Good; 5=Excellent |
|  | Cooperative membership | 0=No; 1=Yes |
|  | Land transfer behavior | 0=No; 1=Yes |
|  | Agricultural income share | Percentage of household income from farming (%) |
|  | Village economic development level | 1=Very low; 2=Low; 3=Medium; 4=High; 5=Very high |
|  | Distance to county seat | Kilometers from village to county center |
| Mechanism Variables | Farmland transfer-in | Transferred-in farmland area, (mu) logarithmic transformation |
|  | Agricultural machinery socialization services | Number of mechanized services adopted (0–6) |
|  | Adoption of green grain production technologies | Number of sustainable practices adopted (0–7) |

**2. Impact Identification Design**

The study employs observational causal inference with the following strategies:

**(1) Model Selection**

Probit Model: For binary outcome cultivation decision (Eq. 1).

Tobit Model: For censored outcome cultivation area (non-adopters = 0) (Eq. 2).

**(2) Identification Assumptions**

Control Variables: Addresses observed confounders (e.g., Land transfer behavior, Village economic development level).

Functional Form: Log-transformation of cultivation area to handle skewness.

Weighted Exposure: Entropy method for digital literacy index reduces measurement error.

Results

The presentation of the results needs clarity.

Section 41. Baseline Regression Results. Why baseline results? How were you able to establish impact from only baseline study?

Thank you for pointing out this! The specific responses are as follows:

**1.Why baseline results?**

The designation “baseline” signifies these results establish the foundational statistical relationship before advanced analyses (e.g., endogeneity tests, robustness checks, Mechanism analyses). This aligns with empirical economics conventions:

**Progressive Modeling:**

Model 1 & 3: Isolate the core effect of farmers’ digital literacy alone on double-cropping rice cultivation decisions/area.

Model 2 & 4: Introduce full control variables to test robustness against confounders (e.g., Land transfer behavior, Village economic development level).

**Benchmark Function:**

Serves as a reference for subsequent rigor tests (IV regression in 4.2, robustness in 4.3) and mechanism studies (4.4).

**2. How “Impact” is Established from Baseline Regressions?**

The study infers impact through three mutually reinforcing strategies:

**(1) Statistical Robustness**

Digital literacy is significant at 5% for decisions (Model 2: coef. = 0.719, p <0.01) and 1% for area (Model 4: coef. = 0.923, p <0.01).

Key controls validate model specification: Land transfer behavior (+), Agricultural income share (+), Village economic development level (+) — all significant with expected signs (Table 3).

**(2) Theoretical Consistency**

Section 4.1 explicitly links results to causal pathways:

“Improved digital literacy increases farmers’ information awareness... facilitates adoption of new technologies... improves market risk management capacity.”

This mirrors the theoretical framework (Fig. 1) and Hypotheses H1-H4.

**(3) Methodological Rigor**

Control for 14 confounders spanning individual/household/village traits (Table 2).

Appropriate models: Probit for binary decisions, Tobit for censored area data.

Large sample: 921 observations ensure statistical power.

**Critical Nuance: Causality Claims**

While baseline results show strong association, the authors do not assert causality from baseline alone:

Explicit acknowledgment of endogeneity risks (Sec 4.2):

“Reverse causality may exist... omitted variable bias is possible.”

Causal validation follows in Sec 4.2 (IV approach) and Sec 4.3 (robustness tests):

IV results (Table 4) confirm digital literacy’s causal effect (coef. = 4.959*** for decisions; 11.385*** for area). Robustness checks (Table 5) sustain significance across model substitutions/re-measurement.

**Conclusion**

“Baseline” appropriately denotes foundational correlational evidence. The term “impact” is contextually justified by:

Theoretical grounding (Sec 2), Statistical robustness (Table 3), Subsequent causal validation (IV/robustness tests).

However, causality is formally established only after addressing endogeneity (Sec 4.2), making the full analysis chain defensible.

The presentation of the results should align with the study objectives/hypothesis.

Thank you for your valuable suggestion! The presentation of results in this study aligns well with the research objectives and hypotheses. Here’s a structured analysis:

**1. Alignment with Research Objectives**

Objective 1 (Assess Impact):

Achieved through Probit/Tobit regression analysis (Table 3) showing digital literacy’s significant positive effect on double-cropping rice adoption (p<0.01) and cultivated area expansion (p<0.01).

Objective 2 (Reveal Mechanisms):

Supported by mechanism analysis (Table 6) demonstrating digital literacy enhances enthusiasm through:

Farmland transfer-in acceleration (coefficient=0.335, p<0.01)

Agricultural machinery service utilization (coefficient=1.088, p<0.01)

Green technology adoption (coefficient=1.562, p<0.001)

Objective 3 (Policy Recommendations):

Directly informed by empirical findings, proposing differentiated strategies based on education levels and off-farm employment status.

**2. Results**

The results section effectively bridges the theoretical framework with empirical evidence. The policy recommendations section closely aligns with mechanism analysis and heterogeneous characteristics, thereby forming a coherent logical chain of “problem identification - mechanism interpretation - precision policymaking”.

6 Further Discussion

This section is out of place. The discussion of the findings should come before the conclusion.

Thank you for pointing out this! we have relocated the discussion of the findings section to precede the conclusion.

Finally, based on the relevant revision suggestions provided by the Reviewers, the variable explanation, theoretical analysis, language expression, and other aspects of the paper were mainly modified and improved. At the same time, the format of the paper was recalibrated and checked. The author made every effort to revise and improve the paper. If there are still areas that have not been fully modified, the editorial department teacher kindly requests forgiveness and proposes revisions. The author will make revisions and improvements at any time.
